# *Lycium barbarum* Polysaccharide Alleviates Renal Damage in Mouse Sepsis by Regulating M1/M2 Macrophage Polarization

**DOI:** 10.1002/fsn3.71561

**Published:** 2026-02-15

**Authors:** Yanfang Zhang, Xiaohang Lu, Hairong Yang, Yuan Zhao, Shanghong Liu

**Affiliations:** ^1^ Medical Record Statistics Department, People's Hospital of Ningxia Hui Autonomous Region Ningxia Medical University Yinchuan Ningxia Hui Autonomous Region China; ^2^ Department of Intensive Care, People's Hospital of Ningxia Hui Autonomous Region Ningxia Medical University Yinchuan China

**Keywords:** *Lycium barbarum* polysaccharide, M1/M2 macrophage polarization, sepsis‐associated kidney injury

## Abstract

*Lycium barbarum* polysaccharide (LBP), a compound derived from the traditional Chinese herb *Lycium barbarum* , has been recognized for its immunomodulatory and anti‐inflammatory properties. Sepsis‐associated acute kidney injury (SA‐AKI) is characterized by systemic inflammation and organ dysfunction. Given the critical role of dysregulated M1/M2 macrophage polarization in the pathogenesis of SA‐AKI, this study investigated the potential protective effects of LBP both in vivo and in vitro. In the mice model of SA‐AKI induced by colonic ligation puncture, LBP administration significantly reduced biochemical indicators of renal injury, including BUN, creatinine, KIM‐1, and NGAL. At the molecular level, LBP decreased the expression of M1 macrophage markers such as iNOS while elevating M2 markers like Arg‐1. Consistent with these findings, experiments in RAW264.7 cells demonstrated that LBP downregulated pro‐inflammatory cytokines IL‐1β, IL‐6, and iNOS, while upregulating anti‐inflammatory IL‐10 and Arg‐1. Further mechanistic analysis revealed that LBP modulates macrophage polarization by regulating the phosphorylation of STAT1 and STAT6, thereby promoting the M2 phenotype and inhibiting M1 activation. Collectively, these results indicate that LBP attenuates SA‐AKI by regulating macrophage polarization.

## Graphical abstract

LBP extracted from *Lycium barbarum* modulates macrophage polarization toward the M2 type and suppresses the M1 type by promoting STAT6 phosphorylation and inhibiting STAT1 phosphorylation. This reduces IL‐1β and IL‐6 while increasing IL‐10, thereby alleviating sepsis‐associated acute kidney injury.

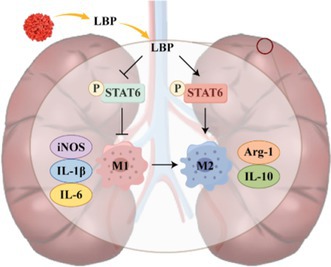

## 1 Introduction

Sepsis is clinically defined as a systemic inflammatory response triggered by infection, which can progress to septic shock and ultimately result in multiple organ failure (Opal and Wittebole 2020; Wiersinga and van der Poll 2022). Kidney ranks as a frequently impacted organ, and the decline in renal function can lead to acute kidney injury associated with sepsis (SA‐AKI), a factor that can exacerbate mortality (Poston and Koyner 2019). SA‐AKI often occurs early in the clinical course and is characterized by a pronounced impairment of the kidney's ability to filter and eliminate nitrogenous waste products. Sepsis accounts for approximately 45%–70% of AKI cases in critically ill patients and is linked to worse outcomes, prolonged hospitalization, and higher mortality rates compared with non‐sepsis‐related AKI (Zarbock, Nadim, et al. 2023). Consequently, SA‐AKI requires greater focus and recognition by scientific researchers.

Current research on the pathophysiology of SA‐AKI focuses primarily on immune‐inflammatory mechanisms (Sun, Chen, et al. 2023). Multiple studies have indicated that macrophage polarization significantly influences the development of SA‐AKI (Juan et al. 2021). There are primarily two types of polarization phenotypes in macrophages: the M1‐like type and the M2‐like type. Exposure to lipopolysaccharide (LPS) and interferon‐gamma (IFN‐γ) drives M1 polarization characterized by transcriptional upregulation of inducible nitric oxide synthase (iNOS) and the release of proinflammatory mediators such as interleukin‐6 (IL‐6), tumor necrosis factor‐alpha (TNF‐α), and interleukin‐1 beta (IL‐1β) (S. Chen et al. 2023). Conversely, stimulation with IL‐4 and IL‐13 promotes M2 polarization, accompanied by elevated expression of arginase‐1 (Arg‐1) and the secretion of anti‐inflammatory cytokines, most notably IL‐10 (S. Chen et al. 2023). During sepsis, M1 macrophages chemotactically migrate to the site of infection and release proinflammatory mediators such as TNF‐α and IL‐1β, exacerbating SA‐AKI tissue damage. In contrast, M2 macrophages secrete IL‐10 and Arg‐1, which inhibit TNF‐α and thereby mitigate renal injury (Zhang et al. 2021). Recent studies have demonstrated that inhibiting macrophage M1 polarization can improve renal injury induced by various mechanisms, including crush syndrome (Li et al. 2022), ischemia–reperfusion injury (Zhou et al. 2024), and sepsis (Chunling et al. 2025). These findings further underscore that modulating macrophage polarization represents a targetable renal protective strategy.

*Lycium barbarum* polysaccharide (LBP) is a principal bioactive component extracted from the traditional Chinese herb *Lycium barbarum* . It is recognized for its potent antioxidant properties, which enhance serum antioxidant markers and protect against oxidative stress induced by chemical or physiological challenges. LBP also exhibits anti‐aging and neuroprotective effects (Zhao et al. 2025). The influence of LBP on human clinical outcomes has been explored. By alleviating ferroptosis, LBP can improve compound‐induced reproductive damage (Yang et al. 2024). LBP ameliorates PM2.5‐induced skin damage, which is related to its antioxidant function (Zhu et al. 2022). It can also mitigate high‐fat diet‐induced sarcopenia (Ren et al. 2025). Notably, accumulating evidence highlights the immunomodulatory and anti‐inflammatory functions of LBP (Fu et al. 2021; Lai et al. 2022; Tian et al. 2019). It demonstrates potent immunomodulatory capabilities across multiple key innate immune cells, including the activation of dendritic cells and the re‐polarization of macrophages (Duan et al. 2024). Specifically, by modulating macrophage polarization, LBP has been shown to mitigate LPS‐induced inflammation, improve inflammatory bowel disease, and suppress breast cancer progression (Ding et al. 2021; Liu et al. 2024; Wang et al. 2023). Given this established role in immunomodulation, LBP represents a promising candidate for the treatment of SA‐AKI. However, its potential therapeutic effect on sepsis‐associated renal injury remains unexplored, and whether LBP can alleviate or prevent SA‐AKI has not yet been elucidated.

This study examined the therapeutic effect of LBP in the SA‐AKI model and elucidated its functional mechanism. Our findings indicate that LBP markedly attenuates renal injury by regulating macrophage polarization and promoting a phenotypic switch toward the M2 anti‐inflammatory state.

## 2 Materials and Methods

### 2.1 Experimental Animals and Cell Lines

This study involved 30 8‐week‐old male C57 mice purchased from Shanghai Model Organisms Center Inc. The mice weighed between 20 and 25 g, with an average weight of 23.04 ± 1.11 g before the start of the experiment. All animal protocols were approved by the Ethical Committee of the People's Hospital of Ningxia Hui Autonomous Region. Mice were maintained in specific pathogen‐free conditions at 18°C–22°C, 50%–60% humidity, 12 h light/12 h dark, with unlimited food and water. Moreover, the RAW264.7 mouse macrophage cell line, which was procured from the Cell Bank of the Chinese Academy of Sciences, was cultured in RPMI‐1640 medium fortified with 10% fetal bovine serum and 1% penicillin–streptomycin, and the cells were kept in an incubator at 37°C under a 5% CO_2_ atmosphere.

### 2.2 Main Reagents and Materials

Isoflurane was purchased from RWD Life Science Co. LBP was purchased from Solarbio Science & Technology Co Ltd. (Cat No. SP9311, China). iNOS (Cat No. 80517‐1‐RR, China), Arg‐1 (Cat No. 16001‐1‐AP, China), F4/80 (Cat No. 29414‐1‐AP, China), and GAPDH (Cat No. 60004‐1‐Ig, China) antibodies were purchased from Proteintech Group Inc. Urea Assay Kit (BUN, Cat No. C013‐2‐1, China), Creatinine Assay kit (Creatinine, Cat No. C011‐2‐1, China), Kidney Injury Molecule 1 Assay Kit (KIM‐1, Cat No. H436‐1‐1, China), and Neutrophil gelatinase‐associated lipocalin Assay Kit (NGAL, Cat No. H392‐1‐1, China) were purchased from Nanjing Jiancheng Bioengineering Institute. Fetal Bovine Serum (FBS, Cat No. A5670701, USA) and Dulbecco's Modified Eagle Medium (DMEM, Cat No. 11965092, USA) were purchased from Thermo Fisher Scientific. Cell Counting Kit‐8 (CCK‐8) and cell lysis buffer for tissues were purchased from Beyotime Biotechnology. The Trizol reagent used for RNA extraction was purchased from Sigma‐Aldrich; the HiScript II Q RT SuperMix kit for reverse transcription and the SYBR Mix for real‐time PCR were obtained from Vazyme Biotech Co. Ltd.

### 2.3 Animal Modeling and Grouping

Thirty male C57BL/6J mice were randomly divided into 5 groups, with 6 mice in each group. Group A was used as a sham operation (Sham group), while groups B, C, D, and E were designated as CLP, CLP + LBP 100 mg/kg, CLP + LBP 200 mg/kg, and CLP + LBP 400 mg/kg groups, respectively.

For CLP, mice were anesthetized by inhalation of 2% isoflurane after 12 h of fasting but free water intake. A ventral midline incision was made to exteriorize the cecum. Subsequently, the cecum was ligated 1 cm from the blind‐ending, and then punctured with a 20‐gauge needle between the ligature and the blind‐ending. The puncture site was gently compressed to extrude a little droplet of fecal contents through the perforation. The cecum was repositioned into the cavity, the peritoneum sutured; the sham‐operation group received identical handling minus ligation and puncture. On the 2 day after surgery, mice in groups C, D, and E were gavaged once with 100, 200, and 400 mg/kg of LBP, respectively. Mice in groups A and B received an equal amount of 1× phosphate buffered saline (PBS) instead of LBP. Blood samples were collected from the mice after 24 h. Following blood collection, the mice were euthanized via intraperitoneal injection of sodium pentobarbital at a dose of 150 mg/kg. Subsequently, renal tissues were harvested.

The research protocol was approved by the Ethical Committee of the People's Hospital of Ningxia Hui Autonomous Region [2022‐NZR‐170]. All experiments were performed in accordance with relevant guidelines and regulations. This study is reported in accordance with ARRIVE guidelines 2.0.

### 2.4 Determination of BUN, Creatinine, KIM‐1, and NGAL Levels

Blood samples were collected from the mice's eyes. After centrifugation at 2000 rpm for 15 min at 4°C, the supernatant was collected. The levels of BUN, creatinine, KIM‐1, and NGAL were measured using the Nanjing Jiancheng Bioengineering Institute kit following the manufacturer's instructions.

### 2.5 HE Staining

Renal histology was evaluated via HE staining. Kidney tissue was fixed in 4% PFA, dehydrated, embedded, sectioned at 5 μm, and counter‐stained with hematoxylin for 3 min. The samples were differentiated using 1% hydrochloric acid alcohol, rinsed in distilled water, counterstained with 0.5% ammonia water, rinsed again, stained with eosin for 1 min, and finally cleared with xylene. Structural and morphological changes in the kidney were examined under a microscope. The renal pathological score was performed in a blinded manner using the semi‐quantitative scoring system corresponding to the degree of renal tubular damage, following a previously published method: 0 = no injury, 1 = 0%–10%, 2 = 11%–25%, 3 = 26%–50%, 4 = 51%–75%, 5 = over 76% (Gao et al. 2025). Five fields were randomly selected from each mouse and the average score was calculated.

### 2.6 Cell Experimental Design

Cell experiments were divided into 5 groups: (A) M0 group; (B) LBP 400 μg/mL group; (C) LPS group; (D) LPS + LBP 200 μg/mL group; (E) LPS + LBP 400 μg/mL group; (F) LPS + LBP 800 μg/mL group. To generate M1 macrophages, RAW264.7 cells were treated with 100 ng/mL LPS for 24 h (Chen et al. 2022). For the polarization of M2 macrophages, the cells were treated with 10 ng/mL IL‐4 and 10 ng/mL IL‐13 for the same duration (Wang et al. 2023). The concurrent processing using LBP and LPS persisted over the span of 24 h. To investigate the mechanism by which LBP regulated macrophage polarization, RAW264.7 cells with different polarization phenotypes were treated with 100 μM fludarabine (MedChemExpress, USA) and 100 nM AS1517499 (MedChemExpress, USA), respectively, according to published studies (Scott et al. 2023).

### 2.7 Cell Viability Assay

The impact of LBP on RAW264.7 cell viability was assessed using a CCK‐8 assay kit. Cells in the logarithmic growth phase were cultured in a 96‐well plate at approximately 5 × 10^3^ cells per well and treated with 200, 400, and 800 μg/mL LBP. After 24 h of incubation at 37°C and 5% CO_2_, the medium was discarded and replaced with fresh medium containing CCK‐8 reagent. Absorbance was measured at 450 nm after a 4‐h reaction.

### 2.8 Western Blot

Kidney tissues or cells were lysed in a chilled tissue lysate with protein phosphatase inhibitors. Proteins were quantified using a BCA assay kit after centrifugation at 4°C and 12,000 rpm for 10 min. Proteins were loaded onto a 10% SDS‐PAGE gel, electrophoresed, transferred onto a PVDF membrane, and blocked with 5% skim milk for 1 h. The membrane was incubated overnight at 4°C with primary antibodies against iNOS (1:1000), Arg‐1 (1:5000), and GAPDH (1:50000). After incubation with HRP‐conjugated goat anti‐rabbit IgG (1:3000) for 60 min at room temperature, the protein was visualized using a chemiluminescence detection kit, and the gray‐scale analysis was performed using Image J software.

### 2.9 Immunofluorescence Staining

Kidney tissue or mouse cells fixed with 4% paraformaldehyde were permeabilized with 0.5% Triton X‐100 at room temperature for 20 min and blocked with 5% BSA for 1 h. The samples were incubated overnight at 4°C with diluted primary antibodies F4/80 (1:200), iNOS (1:200), and Arg‐1 (1:200). After rinsing with PBS, fluorescent secondary antibodies were added and incubated at room temperature for 30 min. After three PBS washes, DAPI was used for nuclear staining, and samples were sealed with an anti‐fluorescence quencher. Images were captured using a fluorescence microscope.

### 2.10 qPCR

The culture medium was removed, and samples were rinsed 2–3 times with PBS. Total RNA was isolated using 1 mL of Trizol reagent, and RNA concentration was measured. cDNA was synthesized from the total RNA and used as a template for reverse transcription. The reaction conditions included initial denaturation at 95°C for 5 min, followed by 40 cycles of denaturation at 95°C for 30 s, annealing at 60°C for 30 s, and extension at 72°C for 20 s. Results were analyzed using the 2^‐ΔΔCT^ method. Table 1 lists the primers used in the study.

**TABLE 1:** Primers used for quantitative PCR.

Gene	Primer	Sequence
iNOS	Forward	TCACTCAGCCAAGCCCTCAC
	Reverse	TCCAATCTCTGCCTATCCGTCTC
Arg‐1	Forward	TGTCCCTATGACAGCTCCTT
	Reverse	GCATCCACCCAATGAACAT
IL‐1β	Forward	CACTACAGGCTCCGAGATGAACAAC
	Reverse	TGTCGTTGCTTGGTTCTCCTTGTAC
IL‐6	Forward	CAGCTGCAGGACGAGATGTGCAA
	Reverse	GCACAGGACTCGACGTTCTGCT
IL‐10	Forward	AATAAGCTCCAAGACCAAGGTGT
	Reverse	CATCATGTATGCTTCTATGCAGTTG

### 2.11 Statistical Analysis

All statistical analyses were performed with GraphPad Prism 8.0 software. Data are presented as mean ± SD unless otherwise stated. Normality was evaluated for each group with the Shapiro–Wilk test. The one‐way ANOVA with Tukey's post hoc test was used for all pairwise comparisons among multiple groups. *p* < 0.05 in every figure representing statistical significance.

## 3 Results

### 3.1 LBP Significantly Reduces Kidney Damage Severity in Septic Mice

Blood urea nitrogen (BUN) is a key indicator of acute kidney injury. Impaired renal excretory function following kidney damage results in elevated serum BUN levels (Zarbock, Weiss, et al. 2023). Creatinine is widely recognized as a diagnostic marker for SA‐AKI, primarily excreted from the bloodstream by the kidneys, and severe renal injury leads to a marked increase in serum creatinine (Cao et al. 2022). Kidney injury molecule‐1 (KIM‐1), an injury‐induced transmembrane glycoprotein, is specifically upregulated in damaged proximal tubular epithelial cells and plays a critical role in mediating processes associated with renal injury (Higgins et al. 2021; Pyo et al. 2021). Similarly, neutrophil gelatinase‐associated lipocalin (NGAL) is a biomarker closely associated with renal function and SA‐AKI (He et al. 2022). In SA‐AKI, NGAL expression abnormally increases in proximal tubular epithelial cells.

**FIGURE 1:**
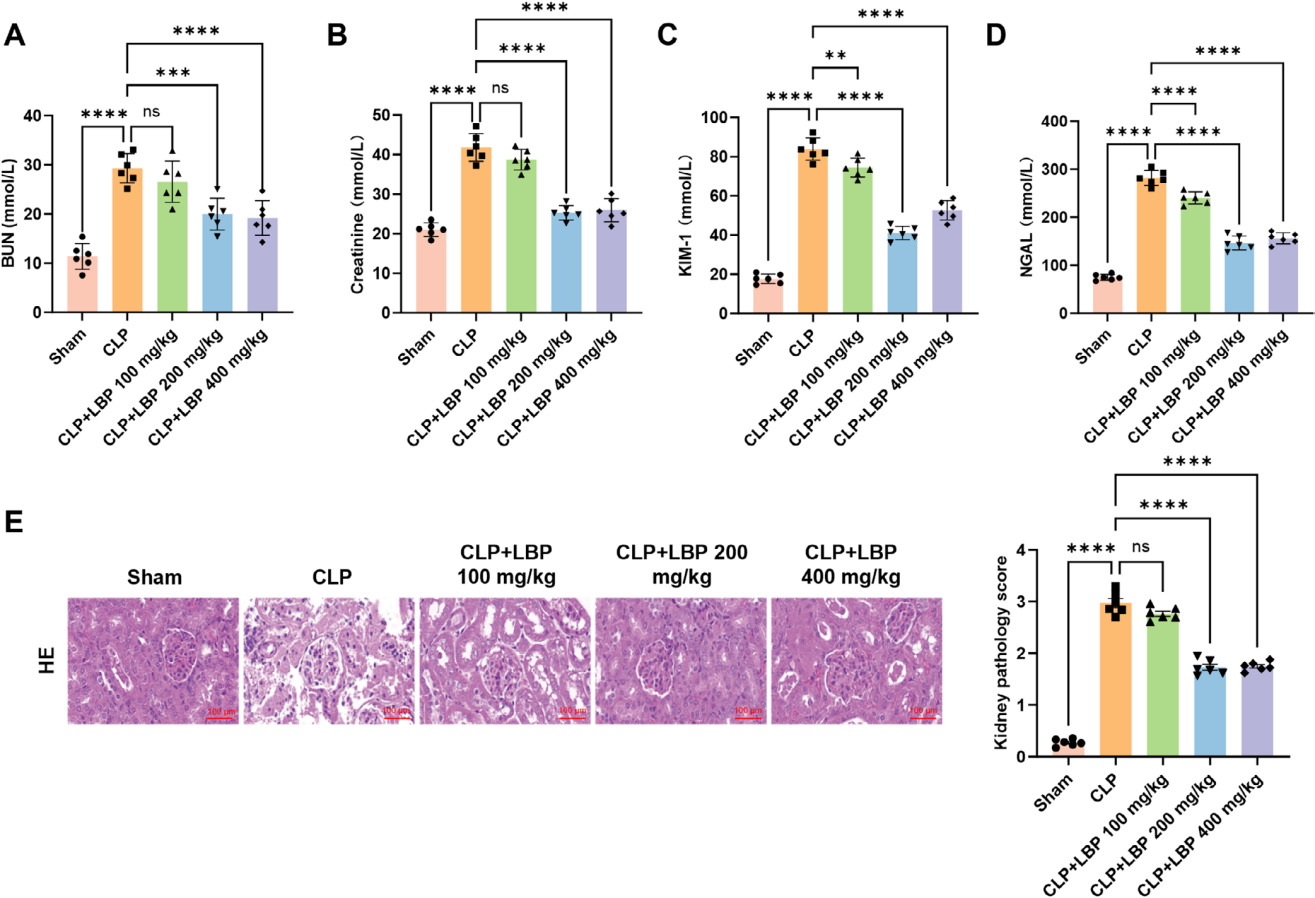
LBP significantly alleviated the severity of CLP‐induced septic kidney injury in C57 mice. (A) Concentrations of BUN found in the blood serum of mice across each group (*n* = 6). (B) Concentrations of creatinine in the blood serum of mice across different groups (*n* = 6). (C) Concentrations of KIM‐1 in the blood serum of mice across different groups (*n* = 6). (D) The concentration of NGAL in the blood serum of mice across each group (*n* = 6). (E) Representative HE in the developmental stages of renal tissue (*n* = 6). **p < 0.01, ***p < 0.001, ****p < 0.0001. ns, no significance.

Experimental results showed that serum levels of BUN, Cr, KIM‐1, and NGAL were significantly elevated in the CLP group compared with the Sham group. Relative to the CLP group, the CLP + LBP 100 mg/kg group showed no significant changes in these indicators. In contrast, both the CLP + LBP 200 mg/kg and CLP + LBP 400 mg/kg groups exhibited a significant decrease in these biomarkers, with the most pronounced reduction observed in the CLP + LBP 200 mg/kg group (Figure 1A–D). Subsequent histological analysis of kidney tissues revealed that the CLP + LBP 200 mg/kg group displayed markedly attenuated renal damage and inflammatory cell infiltration compared to the CLP group (Figure 1E).

### 3.2 LBP Alleviates Mouse Septic Acute Kidney Injury by Regulating Macrophage Polarization In Vivo

**FIGURE 2:**
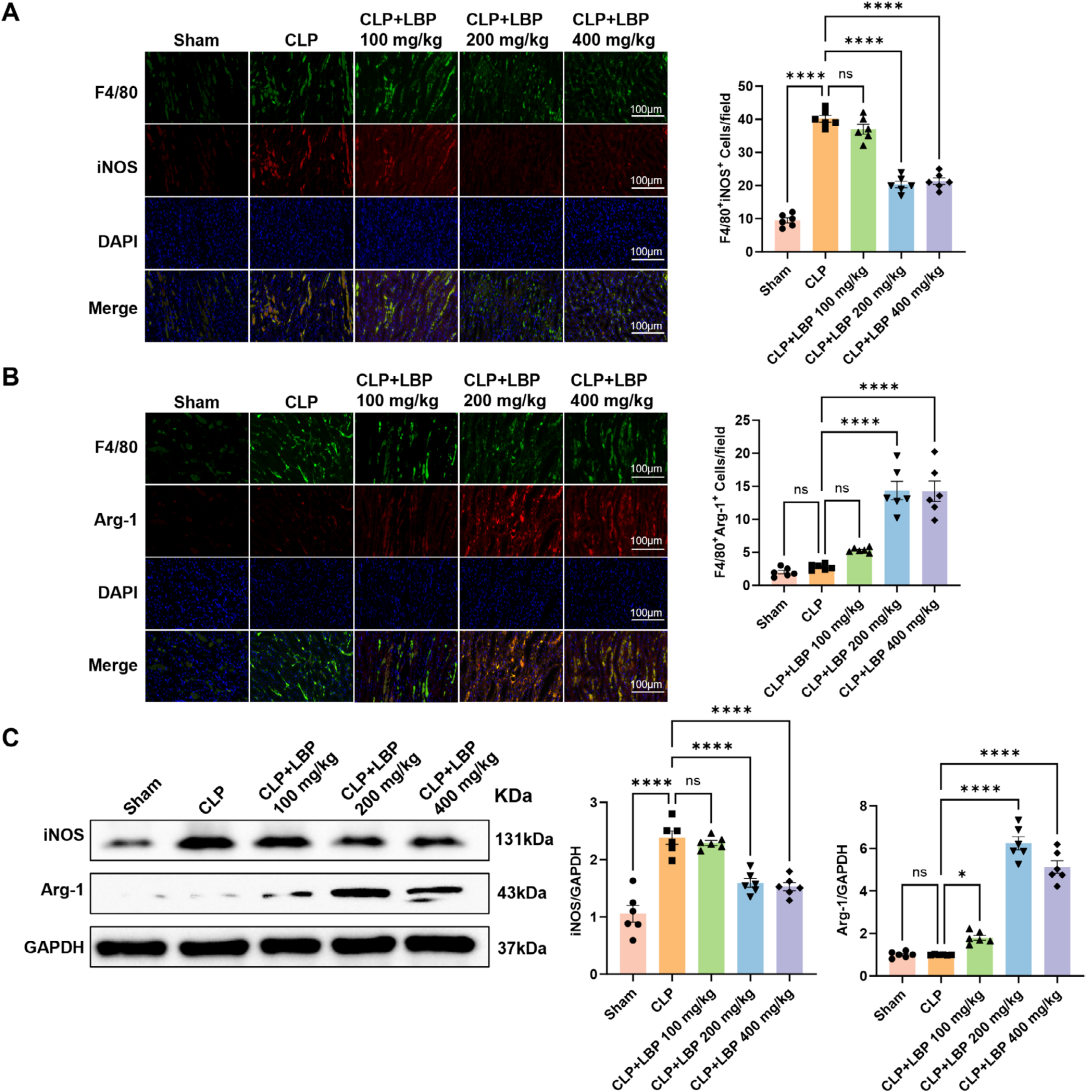
LBP treatment can improve septic acute kidney injury. (A) Exemplary immunofluorescence visuals of F4/80 (green) and iNOS (red) in the renal tissues of the five mouse groups, featuring cell nuclei colored with DAPI (blue) (*n* = 6). (B) Exemplary immunofluorescence visuals of F4/80 (green) and Arg‐1 (red) in the kidney tissues of five mouse groups, featuring cell nuclei colored with DAPI (blue) (*n* = 6). (C) Outcomes from Western blot analysis of iNOS and Arg‐1 in the renal tissues across five mouse groups (*n* = 6). *p < 0.05, ****p < 0.0001. ns, no significance.

In SA‐AKI, renal tubular epithelial cells are exposed to multiple stressors, including ischemia, hypoxia, and inflammatory mediator attacks, leading to rapid declines in kidney function and significant increases in serum creatinine levels (Ostermann et al. 2025). This progression is further aggravated by the polarization of macrophages toward the pro‐inflammatory M1 phenotype, which promotes ferroptosis in tubular epithelial cells (Zhang et al. 2024). To further investigate whether LBP attenuates SA‐AKI through macrophage polarization regulation, immunofluorescence double staining was performed. The results revealed a significant decrease in F4/80^+^iNOS^+^ macrophages in SA‐AKI mouse tissues after LBP treatment, with the most pronounced effect observed at the 200 mg/kg dose. Concurrently, LBP treatment at 200 mg/kg led to a notable increase in F4/80^+^Arg‐1^+^ macrophages. These findings collectively support the hypothesis that LBP may alleviate SA‐AKI by shifting macrophage polarization from the M1 to the M2 phenotype (Figure 2A,B). Consistent with these findings, western blot analysis demonstrated that iNOS protein levels were significantly lower, while Arg‐1 expression was higher in the CLP + LBP 200 mg/kg group compared with the CLP‐alone group (Figure 2C).

### 3.3 LBP Alleviates Septic Kidney Injury In Vitro by Regulating Macrophage Polarization

**FIGURE 3:**
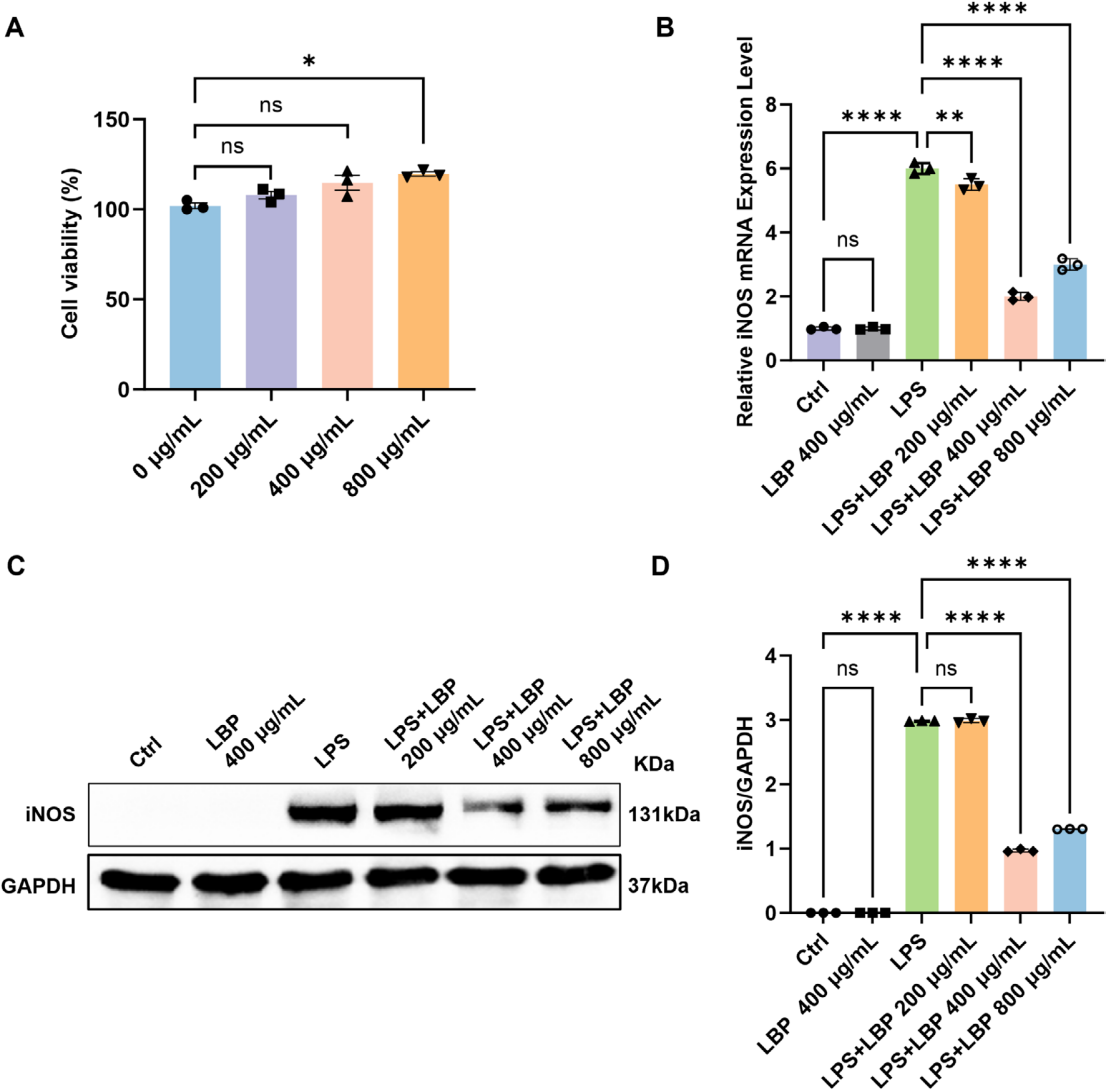
The effect of LBP on the viability of RAW264.7 cells and the optimal LBP concentration for macrophage polarization. (A) RAW264.7 cells underwent cultivation with varied quantities of LBP over a 24‐h period to determine their viability (*n* = 3). (B) Results of qPCR for iNOS (*n* = 3). (C, D) Analysis via Western blotting of iNOS protein amounts in RAW264.7 cells exposed to LBP and LPS over a 24‐h period (*n* = 3). *p < 0.05, **p < 0.01, ****p < 0.0001. ns, no significance.

**FIGURE 4:**
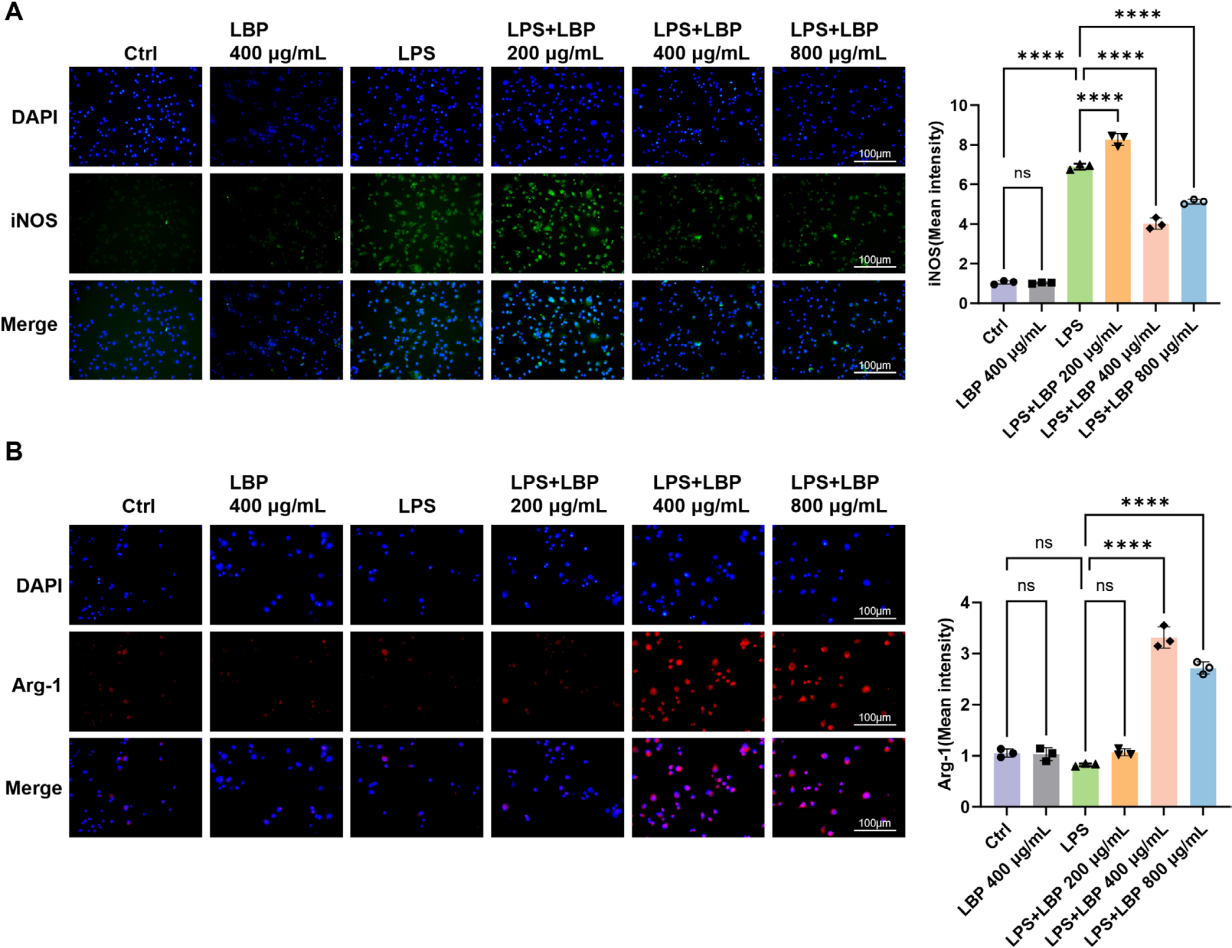
Observation of the effects of LPS on iNOS and Arg‐1 expression in RAW264.7 cells using immunofluorescence staining. (A) Different groups exhibit immunofluorescence staining of iNOS (red) (*n* = 3). (B) Different groups of Arg‐1 (green) immunofluorescence staining (*n* = 3). ****p < 0.0010. ns, no significance.

To further investigate the mechanism by which LBP modulates macrophage polarization, we performed in vitro experiments using the RAW264.7 cell line. The cell viability was assessed using a CCK‐8 assay, and the results showed that LBP at concentrations of 200, 400, and 800 μg/mL had no toxic effects on RAW264.7 cells (Figure 3A). We then examined the effect of LBP on M1 polarization markers in LPS‐stimulated RAW264.7 cells. Quantitative PCR results showed a significant decrease in iNOS mRNA levels at 400 μg/mL LBP after LPS stimulation, whereas LBP alone had no effect, indicating that its activity is specific to the regulation of polarization rather than baseline cell function (Figure 3B). This finding was corroborated at the protein level by western blot, where 400 μg/mL LBP most effectively suppressed iNOS expression (Figure 3C,D). Immunofluorescence staining further validated the polarization shift. LPS stimulation markedly increased iNOS expression, which was substantially reversed by 400 μg/mL LBP (Figure 4A). Conversely, Arg‐1 expression was downregulated by LPS but was restored upon co‐treatment with 400 μg/mL LBP (Figure 4B). Hence, 400 μg/mL LBP was chosen as the working dose for follow‐up cell‐based experiments.

### 3.4 LBP Inhibits M2 to M1 Reprogramming in RAW264.7 Cells

**FIGURE 5:**
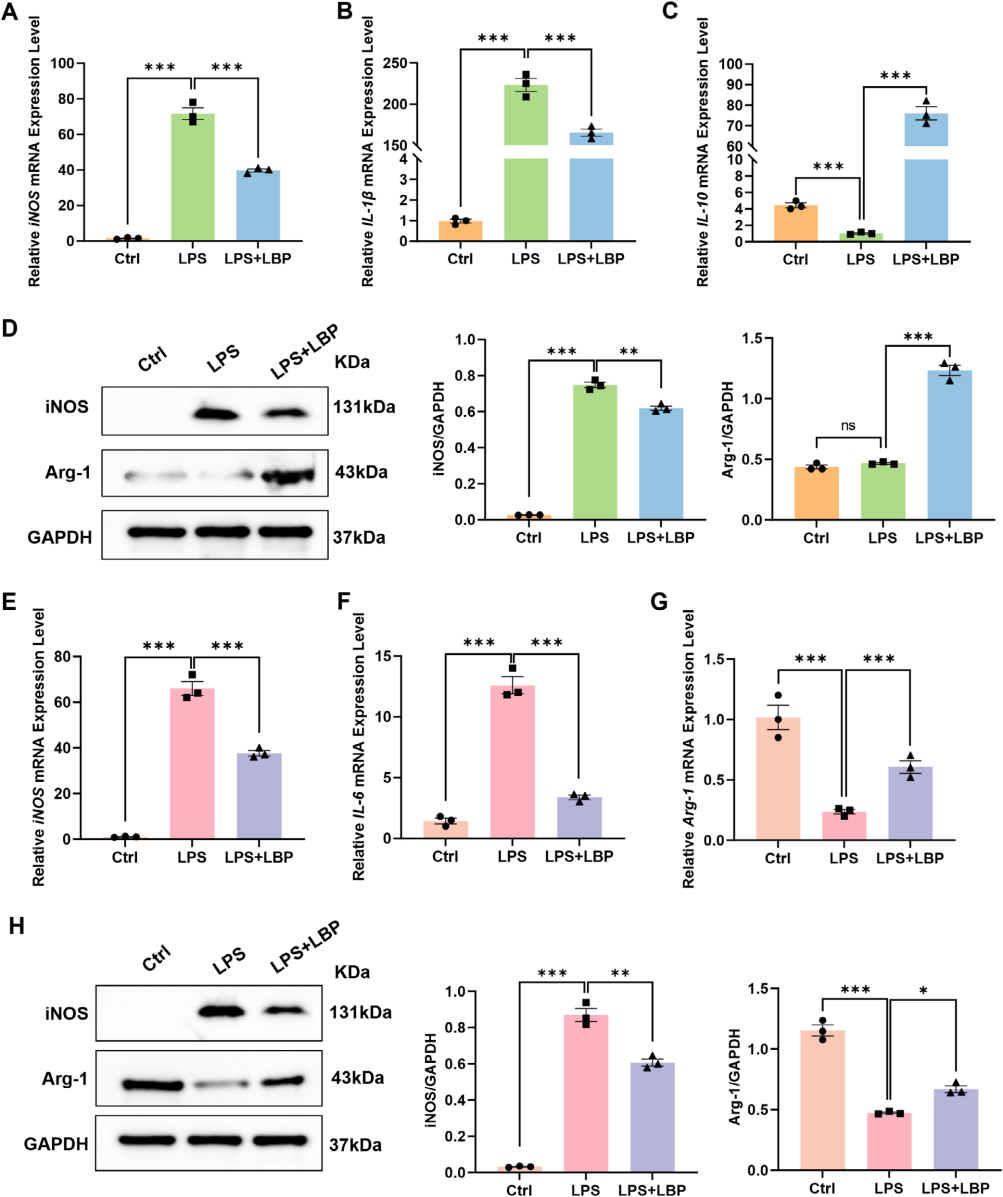
Using qPCR and Western blot to detect the effects of LPS on the expression of macrophage polarization‐related genes. (A–C) Outcomes from qPCR for iNOS, IL‐1β, and IL‐10 in groups Ctrl (M0), LPS, and LPS + LBP (*n* = 3). (D) Results of the Western blot for iNOS, Arg‐1, and GAPDH across the Ctrl (M0), LPS, and LPS + LBP groups (*n* = 3). (E–G) Outcomes of qPCR for iNOS, IL‐6, and Arg‐1 across the Ctrl (M2), LPS, and the LPS + LBP groups (*n* = 3). (H) Outcomes of Western blot analysis for iNOS, Arg‐1, and GAPDH among the Ctrl (M2), LPS, and LPS + LBP cohorts (*n* = 3). *p < 0.05, **p < 0.01, ***p < 0.001.

To evaluate the regulatory effect of LBP on macrophage polarization, we measured the expression of M1 and M2 markers in RAW264.7 cells at both mRNA and protein levels. Following LPS stimulation, mRNA levels of the M1 markers iNOS and IL‐1β were significantly elevated, which was markedly attenuated by LBP co‐treatment (Figure 5A,B). Conversely, the expression of the anti‐inflammatory cytokine IL‐10 was suppressed by LPS but was restored to a significantly higher level upon LBP administration (Figure 5C). Western blot analysis results were consistent, showing that LPS stimulation increased iNOS protein expression and decreased Arg‐1, while LBP treatment reversed these changes (Figure 5D).

We next induced an M2 phenotype in RAW264.7 cells using IL‐4 and IL‐13, and exposed the cells to LPS with or without LBP. Quantitative PCR analysis revealed that LPS stimulation of M2 cells significantly upregulated iNOS and IL‐6 mRNA while downregulating Arg‐1. When treated with LBP, the induction of iNOS and IL‐6 was markedly suppressed, and Arg‐1 expression was restored (Figure 5E–G). The corresponding results were confirmed by Western blotting (Figure 5H). Thus, these findings indicate that LBP can prevent the repolarization of RAW264.7 cells from M2 to M1, aiding in maintaining an M2‐like phenotype.

### 3.5 LBP Regulated STAT1 and STAT6 Phosphorylation to Maintain the M2 Phenotype and Suppress the M1 Phenotype

**FIGURE 6:**
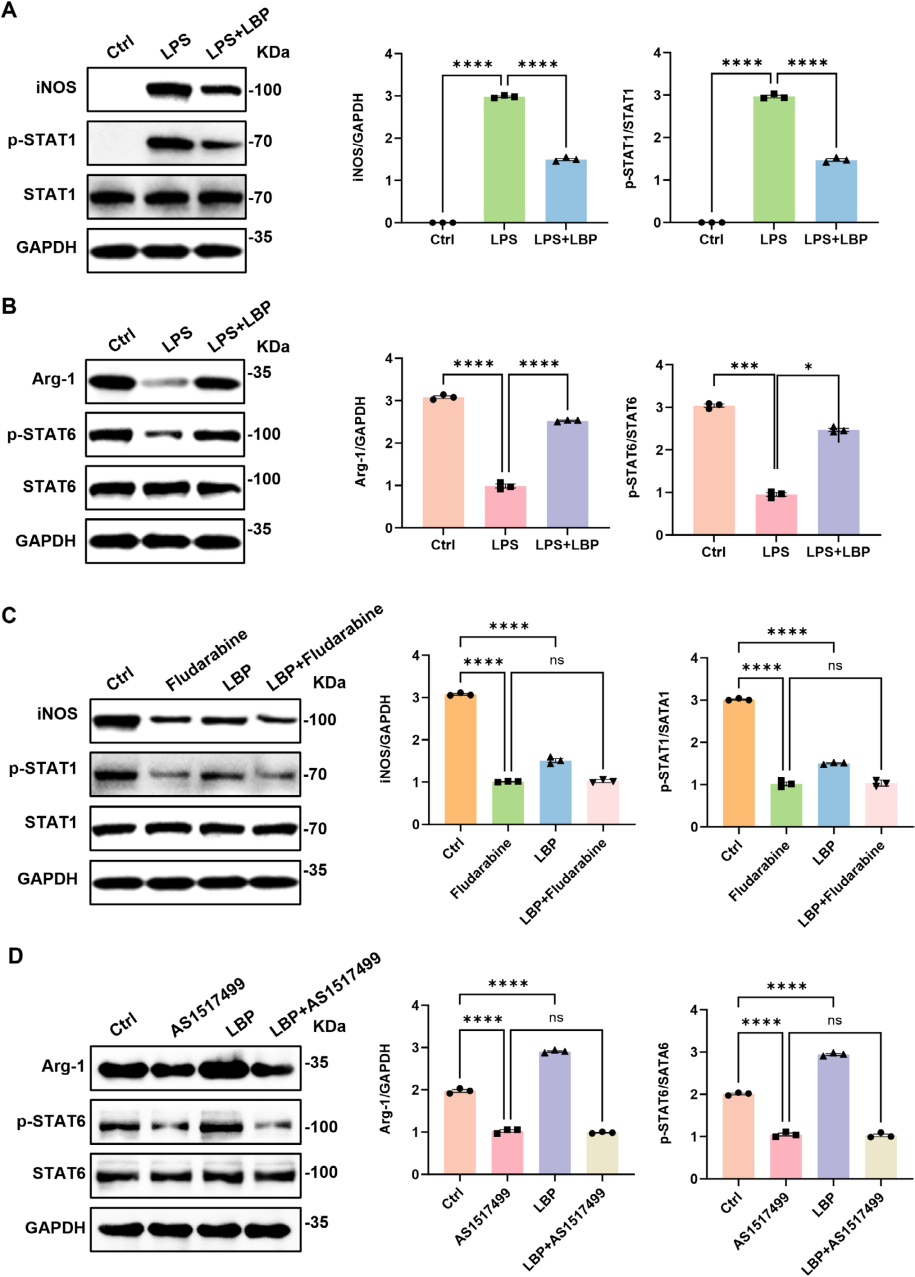
Using Western blot to detect the effects of LPS on macrophage polarization mediated by STAT1/STAT6 phosphorylation. (A) Results of the Western blot for iNOS, p‐STAT1, STAT1 and GAPDH across the Ctrl (M0), LPS, and LPS + LBP groups (*n* = 3). (B) Results of the Western blot for Arg‐1, p‐STAT6, STAT6 and GAPDH across the Ctrl (M2), LPS, and LPS + LBP groups (*n* = 3). (C) Western blot results of iNOS, p‐STAT1, STAT1 and GAPDH across the Ctrl (M1), LBP and/or Fludarabine groups (*n* = 3). (D) Western blot results of Arg‐1, p‐STAT6, STAT6 and GAPDH across the Ctrl (M2), LBP and/or AS1517499 groups (*n* = 3). *p < 0.05, ***p < 0.001, ****p < 0.0001, ns, no significance.

It is well established that macrophage polarization is a complex process regulated by diverse mechanisms and signaling pathways, in which transcription factors play indispensable roles. Specifically, STAT1 and STAT6 are recognized as critical regulators of M1 and M2 polarization, respectively (Yan et al. 2024). In detail, STAT1 is primarily activated by pro‐inflammatory signals such as LPS, driving the expression of iNOS, IL‐12, and TNF‐α (Zhou et al. 2020). Conversely, STAT6 is phosphorylated and activated upon stimulation with IL‐4 or IL‐13, inducing the expression of Arg‐1, Fizz1, and Ym1. Previous studies have confirmed that LBP modulated the phosphorylation of both STAT1 and STAT6 (Zhou et al. 2020). To further validate this, we conducted subsequent experiments. As shown in Figure 6A,B, LBP inhibited LPS‐induced phosphorylation of STAT1 while promoting the expression of p‐STAT6, accompanied by decreased iNOS and increased Arg‐1 expression. Furthermore, using the STAT1 inhibitor fludarabine significantly suppressed STAT1 phosphorylation, and combining it with LBP did not produce additional inhibition, indicating that LBP acts through the STAT1 pathway to restrain M1 polarization (Figure 6C). Similarly, we found that LBP enhanced STAT6 phosphorylation, and this effect was abolished when combined with the STAT6 inhibitor AS1517499, suggesting that LBP sustains M2 macrophage polarization via STAT6 phosphorylation (Figure 6D).

## 4 Discussion

SA‐AKI is a frequent and severe clinical complication that substantially threatens patient health, elevates the risk of chronic comorbidities, and is associated with high mortality (Peerapornratana et al. 2019). The pathogenesis of SA‐AKI remains incompletely understood. Notably, dysregulated macrophage polarization has been identified as a central driver of disease progression (Kadomoto et al. 2021). Given the clinical significance of SA‐AKI and the pivotal role of macrophage imbalance, the development of safe and effective therapeutic agents targeting this mechanism represents an urgent unmet need.

The bioactive component LBP derived from the traditional Chinese herb *Lycium barbarum* exhibits a spectrum of pharmacological properties, including anti‐inflammatory, antioxidant, and immunomodulatory activities (Qi et al. 2022; Sun, Du, et al. 2023). Previous studies have demonstrated that LBP mitigates inflammation caused by LPS through inducing the degradation of pyruvate kinase M2 (PKM2) and modulating macrophage differentiation (Ding et al. 2021). In IBD, LBP inhibits M1 polarization via suppression of STAT1 signaling while promoting M2 polarization through enhanced STAT6 activation (Wang et al. 2023). Notably, LBP appears to have two opposite effects on macrophage polarization, and LBP promotes M1 polarization in breast cancer, which has been shown to be beneficial in inhibiting cancer progression (Liu et al. 2024). To investigate the potential of LBP in SA‐AKI, a kidney injury model was created in mice to replicate human kidney disease symptoms. Our results show that LBP treatment significantly reduced established renal injury markers, such as BUN, creatinine, urinary KIM‐1, and NGAL transport protein, and attenuated histopathological damage in the kidney.

Numerous research studies suggested that M1 and M2 type macrophages promoted SA‐AKI by influencing the concentration of inflammatory factors (Juan et al. 2021). Our analysis of macrophage polarization in kidney‐damaged mice revealed that LBP therapy enhanced their polarization to an M2 type and suppressed the M1‐like state of macrophages. These results were also confirmed in RAW264.7 cells. Remarkably, the in vivo treatment group administered 200 mg/kg LBP demonstrated the most pronounced protective effect, whereas in vitro, the equivalent dose was 400 μg/mL. In fact, this significant discrepancy between in vivo and in vitro doses has been documented in relevant studies. In a murine colitis model, 200 mg/kg LBP significantly promoted M2 polarization in tissues of the colon from mice, whereas in cells, 400 μg/mL yielded optimal effects (Wang et al. 2023). This duality may be related to the physical properties of polysaccharides. LBP is a highly polar macromolecule whose oral absorption is negatively impacted by its molecular weight, and most plant polysaccharides exhibit low bioavailability (Gan et al. 2020). The concentrations of 100 mg/kg of LBP detected in plasma and kidney after 24 h of oral administration were less than 5 μg/mL and 10 μg/g, much lower than the administered concentrations, which may explain this in vivo and in vitro dose difference (Xia et al. 2021).

Research demonstrates that the polarization phenotype of macrophages can be reversed by varying chemokine conditions, leading to a total transformation from an M2 to an M1 phenotype (Das et al. 2015). To further investigate the mechanism by which LBP modulates macrophage polarization, we specifically examined its role during the repolarization of M2 macrophages. After inducing an M2 phenotype with IL‐4/IL‐13, subsequent stimulation with LPS prompted a shift toward an M1‐like state. However, LBP treatment effectively inhibited this repolarization, helping to maintain an M2 phenotype. More importantly, we have confirmed that LBP can affect macrophage polarization by regulating the phosphorylation of STAT1/STAT6, which is consistent with previous research findings (Wang et al. 2023). In addition, to exclude the potential influence of LBP itself on the baseline status of macrophages, a control group treated with LBP alone (400 μg/mL) was included. The results showed that, in the absence of LPS stimulation, LBP treatment did not significantly alter the expression of M1 or M2 macrophage markers. This indicates that LBP does not directly interfere with the polarization balance of macrophages under resting conditions, further supporting the conclusion that LBP exerts its immunoregulatory function by influencing STAT1/STAT6 phosphorylation in activated macrophages.

Our findings revealed that LBP suppresses the polarization of M1 macrophages while enhancing it in M2 macrophages. Our validation of these findings was conducted in vitro and in vivo, respectively, revealing that the group treated with LBP exhibited elevated expression rates of IL‐10 and Arg‐1, suggesting that LBP may control the polarization in the M2 phenotype. However, there are some limitations of our study. This study relied on a single animal model and cell line, and the effect of LBP in different animal models or macrophage cell lines needs to be further confirmed. Second, at the mechanistic level, although we demonstrated that LBP could affect macrophage polarization by modulating STAT1/STAT6 phosphorylation, the specific mechanism needs to be further investigated. In addition, our protocol was a single LBP administration, and the effect on continuous or multiple administrations remains to be explored.

To sum up, LBP addresses SA‐AKI through the regulation of macrophage polarization. The research offers fresh insights into how LBP governs acute renal damage and lays a conceptual groundwork for creating LBP as a treatment for SA‐AKI.

## Author Contributions

Yanfang Zhang designed research concept. Yanfang Zhang , Xiaohang Lu and Hairong Yang conducted experiments and drafted the manuscript; Yuan Zhao analyzed the data; Shanghong Liu and Xiaohang Lu provided technical support. Yanfang Zhang supervised the study and edited the manuscript. The authors read and approved the final manuscript.

## Funding

This work was supported by Ningxia Natural Science Foundation (Project number: 2023AAC03482 and 2024AAC03499).

## Disclosure

No AI was utilized at any stage during research development and design, data collection, manuscript preparation etc.

## Ethics Statement

The research protocol was approved by the Ethical Committee of the People's Hospital of Ningxia Hui Autonomous Region [2022‐NZR‐170].

## Conflicts of Interest

The authors declare no conflicts of interest.

## Data Availability

The original contributions presented in the study are included in the article; further inquiries can be directed to the corresponding author.
